# Trends in Delirium and New Antipsychotic and Benzodiazepine Use Among Hospitalized Older Adults Before and After the Onset of the COVID-19 Pandemic

**DOI:** 10.1001/jamanetworkopen.2023.27750

**Published:** 2023-08-07

**Authors:** Christina Reppas-Rindlisbacher, Alexa Boblitz, Robert A. Fowler, Lauren Lapointe-Shaw, Kathleen A. Sheehan, Therese A. Stukel, Paula A. Rochon

**Affiliations:** 1 Women’s Age Lab and Women’s College Research Institute, Women’s College Hospital, Toronto, Ontario, Canada; 2 Division of General Internal Medicine and Geriatrics, Sinai Health System and the University Health Network, Toronto, Ontario, Canada; 3 Department of Medicine, University of Toronto, Toronto, Ontario, Canada; 4 Institute of Health Policy, Management and Evaluation, University of Toronto, Toronto, Ontario, Canada; 5 ICES, Toronto, Ontario, Canada; 6 Sunnybrook Research Institute, Sunnybrook Health Sciences Centre, Toronto, Ontario, Canada; 7 Centre for Mental Health, University Health Network, Toronto, Ontario, Canada; 8 Department of Psychiatry, University of Toronto, Toronto, Ontario, Canada

## Abstract

**Importance:**

The COVID-19 pandemic caused large disruptions to health care for hospitalized older adults. The incidence and management of delirium may have been affected by high rates of COVID-19 infection, staffing shortages, overwhelmed hospital capacity, and changes to visitor policies.

**Objective:**

To measure changes in rates of delirium and related medication prescribing during the COVID-19 pandemic among hospitalized older adults.

**Design, Setting, and Participants:**

This population-based, repeated cross-sectional study used linked databases to measure rates of delirium and related medication prescriptions among adults aged 66 years or older hospitalized before and during the COVID-19 pandemic (January 1, 2017, to March 31, 2022) in Ontario, Canada.

**Exposure:**

The first 2 years of the COVID-19 pandemic (March 1, 2020, to March 31, 2022).

**Main Outcomes and Measures:**

The main outcomes were weekly rates of delirium per 1000 admitted population and monthly rates of new antipsychotic and benzodiazepine prescriptions per 1000 discharged population. Observed rates were compared with projected rates based on modeling from 3 years before pandemic onset.

**Results:**

Among 2 128 411 hospitalizations of older adults over the 5-year study period (50.7% female; mean [SD] age, 78.9 [8.3] years), absolute rates of delirium increased from 35.9 per 1000 admitted population during the prepandemic period to 41.5 per 1000 admitted population throughout the pandemic. The adjusted rate ratio (ARR) of delirium during the pandemic compared with the projected rate was 1.15 (95% CI, 1.11-1.19). Monthly rates of new antipsychotic prescriptions increased from 6.9 to 8.8 per 1000 discharged population and new benzodiazepine prescriptions from 4.4 to 6.0 per 1000 discharged population and were significantly higher during the pandemic compared with projected rates (antipsychotics: ARR, 1.28; 95% CI, 1.19-1.38; benzodiazepines: ARR, 1.37; 95% CI, 1.20-1.57). Rates were highest during pandemic waves 1 (March to June 2020), 3 (March to June 2021), and 5 (December 2021 to February 2022) and remained elevated above projected levels throughout the first 2 years of the pandemic.

**Conclusions and Relevance:**

In this repeated cross-sectional study of hospitalized older adults, there was a temporal association between COVID-19 pandemic onset and significant increases in rates of delirium in the hospital and new antipsychotic and benzodiazepine prescriptions after hospital discharge. Rates remained elevated over 2 years. Pandemic-related changes such as visitor restrictions, staff shortages, isolation practices, and reduced staff time at the bedside may have contributed to these trends.

## Key Points

**Question:**

Was the COVID-19 pandemic associated with changes in rates of delirium and new dispensing of antipsychotics and benzodiazepines at hospital discharge among hospitalized older adults?

**Findings:**

In this cross-sectional study of 2 128 411 acute care hospitalizations among adults aged 66 years or older from 2017 to 2022, the COVID-19 pandemic was associated with significant increases in rates of delirium and new use of antipsychotics and benzodiazepines after hospital discharge compared with projected trends.

**Meaning:**

These findings may reflect disruptions from staffing shortages, visitor restrictions, and isolation practices.

## Teaser

This cross-sectional study compares rates of delirium and new antipsychotic and benzodiazepine prescriptions before and during the COVID-19 pandemic among hospitalized patients aged 66 years or older in Ontario, Canada.

## Introduction

Nearly 30% of hospitalized older adults experience delirium, a condition that has serious consequences, including increased risk of dementia, institutionalization, and mortality.^1,2^ Delirium incidence may have increased during the COVID-19 pandemic in association with disruptions in usual hospital care and because it was a common complication of COVID-19 among older adults.^3^ The onset, recognition, and management of delirium may have been affected by overwhelmed hospital capacity, staff shortages, isolation procedures, and reduced contact with friends and family due to visitor restrictions.^4^ These factors may have been associated with increased use of medications, such as antipsychotics and benzodiazepines, as staff were unable to provide nonpharmacologic interventions known to be effective for delirium prevention and management.^5,6^

Studies investigating pandemic-related changes in delirium rates and antipsychotic use have yielded mixed results, and to our knowledge, trends have not been described beyond the initial stages of the pandemic. A study across 14 US hospitals demonstrated no significant difference in delirium before and after pandemic onset, but data collection was not continued past June 2020.^7^ One study done in a Japanese emergency department showed an increase in delirium, but data collection was stopped 4 months into the pandemic.^8^ Similarly, a Canadian study of in-hospital antipsychotic use showed an initial increase in March 2020 (from 27.1% to 30.8%), but prescribing returned to prepandemic levels within 8 weeks and subsequent pandemic waves were not studied.^9^ These studies called for future research with a longer time frame to better understand the consequences of the pandemic for delirium incidence and management.

We performed a longitudinal, population-based study to compare the rates of delirium and prescription of a new antipsychotic or benzodiazepine after discharge among hospitalized older adults before and during the pandemic. As a secondary aim, we estimated how those comparisons varied across sociodemographic groups.

## Methods

### Study Design and Setting

We conducted a population-based, repeated cross-sectional study of rates of delirium and newly dispensed antipsychotics and benzodiazepines at hospital discharge in Ontario, Canada, using linked health and administrative data sets (details in the eAppendix in Supplement 1). Ontario is Canada’s most populous province, with more than 14 million adults who are covered for hospital care and physicians’ services under the Ontario Health Insurance Plan. The administrative data sets used in this study are housed at ICES (formerly the Institute of Clinical and Evaluative Sciences). ICES is an independent, nonprofit research institute for which legal status under privacy law allows it to collect and analyze health care and demographic data without consent for health system evaluation and improvement. Our study was authorized under §45 of Ontario’s Personal Health Information Protection Act,^10^ which made it exempt from research ethics board review and informed consent. We reported all methods and outcomes according to best practices described in the Reporting of Studies Conducted Using Observational Routinely-Collected Data (RECORD) statement, an extension of the Strengthening the Reporting of Observational Studies in Epidemiology (STROBE) reporting guideline.^11^

### Data Sources

Patient characteristics, prescription drug use, covariate information, and outcome data were obtained from administrative health databases at ICES (eTable 1 in Supplement 1). We used demographic information from the provincial health insurance registry to measure age, sex, socioeconomic status (income quintiles), and rural location of residence (rurality index for Ontario score >40).^12^ We used a 5-year lookback period to measure 17 high-impact chronic conditions^13,14^ and categorized the number of conditions from 0 to 5 or more (eTable 2 in Supplement 1). Residence in a nursing home was determined using an algorithm based on inclusion in the Continuing Care Reporting System database, medication claims from the Ontario Drug Benefit database, and physician visits from the Ontario Health Insurance Plan (OHIP) database.^15^ Immigrant status was obtained based on presence of a record in the provincial portions of Immigration, Refugees and Citizenship Canada’s Permanent Resident Data. A diagnosis of COVID-19 was defined by any hospital admission with a relevant *International Statistical Classification of Diseases and Related Health Problems, Tenth Revision (ICD-10)* or OHIP billing code.^16,17^

### Study Cohort

We included all acute care hospital admissions for older adults (≥66 years of age) in Ontario between January 1, 2017, and March 31, 2022. We limited the study to patients aged 66 years or older to allow a 1-year lookback period for incident medications using the Ontario Drug Benefit, which covers medication claims for patients aged 65 years or older. We excluded those without provincial health insurance coverage, with missing age or sex data, and older than 105 years. We excluded admissions for same-day surgeries as these procedures are associated with low delirium risk, and we excluded admissions longer than 364 days to limit inclusion of patients with complex continuing care needs. We excluded patients with a diagnosis of schizophrenia using a validated coding algorithm^18^ over a 10-year lookback period to limit the possibility that antipsychotics were prescribed for this indication. From the cohort of overall admissions, we created a separate cohort of patients who survived to discharge to measure the postdischarge medication outcomes.

### Study Exposure

The exposure period was the first 2 years of the COVID-19 pandemic, from March 1, 2020, to the end of complete data availability on March 31, 2022. The nonexposure period was the 3 years before the pandemic to allow for stability in pre–COVID-19 rates of hospitalization and delirium. The exposure period included 5 pandemic waves, defined as months of increased COVID-19 case counts with an effective reproduction number, *R*, significantly greater than 1.^19^ In Ontario, wave 1 was from March to June 2020; wave 2, from September 2020 to February 2021; wave 3, from March to June 2021 (owing to the Alpha variant); wave 4, from September to November 2021 (owing to the Delta variant); and wave 5, from December 2021 to February 2022 (owing to the Omicron variant).^20,21^

### Study Outcomes

Our primary outcome was in-hospital delirium, defined using *ICD-10* codes for delirium (eTable 3 in Supplement 1), which have been shown to be highly specific but weakly sensitive (specificity, 98%; sensitivity, 18%).^22^ The *ICD-10* codes have higher sensitivity for severe delirium (40.0%) and hyperactive or mixed delirium (42.9%) than for hypoactive delirium (14.3%).^22^ Delirium codes were included for any diagnosis type (main, secondary, or postadmission comorbidity) and may have reflected delirium present at hospital admission or hospital-acquired delirium. Our secondary outcomes were new antipsychotic and benzodiazepine prescriptions dispensed within 7 days of hospital discharge, as we did not have access to inpatient medications. New medications were defined using a 1-year lookback period to identify previous outpatient prescriptions, which in Ontario include medications dispensed in nursing homes and hospice. Only antipsychotics commonly used for delirium were included in our secondary outcome (eTable 4 in Supplement 1). There was no minimum dispensed quantity required to define prior medication use or new use after discharge. The 7-day period after discharge has been used in prior studies to identify new prescriptions that were first initiated in hospital or at the time of hospital discharge.^23,24^ If patients were discharged from acute care to another inpatient setting such as inpatient rehabilitation, the 7-day period commenced after discharge to an outpatient setting (eg, community, nursing home).

### Statistical Analysis

Standardized differences were used to compare the balance in baseline characteristics between the periods before and after the onset of the COVID-19 pandemic, with a threshold of greater than 10% representing a meaningful difference.^25^ We expressed delirium rates as the number of people with delirium per 1000 eligible admitted population and antipsychotic and benzodiazepine rates as total dispensed prescriptions per 1000 eligible discharged population. We used Poisson generalized estimating equations for clustered count data to model trends in outcome rates in the pre–COVID-19 period and used these to estimate trends after the onset of the pandemic, as in previous work.^26^ The unit of analysis was the age group, sex, and week stratum for delirium and age group, sex, and month stratum for medications. Monthly strata were used for medications because the weekly new prescribing rate was low. The dependent variable was the count of events in the population in the stratum; the offset was the log of the stratum-specific population, and a first-order autoregressive working correlation structure was used to account for correlations of stratum-specific event rates across time (weeks or months). The pre–COVID-19 models included age group and sex; a linear term of weeks (or months) since January 1, 2017, to estimate secular trends; and pre–COVID-19 month to model seasonal variations, with April as the reference month.

We estimated expected delirium and medication rates after the onset of the pandemic (and 95% CIs) by applying the linear combination of pre–COVID-19 regression coefficients to the postpandemic age group, sex, and month strata and exponentiating. We expressed the relative change in postpandemic onset rates as an adjusted rate ratio (ARR) of observed to projected rates by exponentiating the difference of observed and projected postpandemic onset log rates and 95% CIs.

#### Subgroup Analysis

We repeated analyses separately for the following subgroups relevant to health equity: sex, age at hospital admission (66-74, 75-84, or ≥85 years), income quintile, nursing home residency, rurality, and immigrant status. The purpose of subgroup analysis was not to provide statistical evidence of effect modification but to report the estimates within strata of interest.

#### Sensitivity Analysis

We repeated these comparisons excluding hospital admissions with a diagnosis of COVID-19 to separate the association with pandemic disruptions from COVID-19. Statistical analyses were conducted with SAS, version 9.4 (SAS Institute), and all statistical tests used 2-sided *P* < .05 as the threshold for significance.

## Results

The study sample included 1 047 680 older adults with 2 128 411 hospital admissions between 2017 and 2022 (mean [SD] age, 78.9 [8.3] years; 50.7% female; 49.3% male). The median length of stay was 5 days (IQR, 2-9 days), and 6.3% of patients were discharged to an inpatient rehabilitation facility. The cohort creation flow diagram is shown in the eFigure in Supplement 1. Characteristics of admitted older adults, presented by year of admission in the Table, were similar (standardized differences, <10%) before (January 1, 2017, to February 29, 2020) and after (March 1, 2020, to March 31, 2022) the onset of the pandemic.

**Table.** Demographic Characteristics of Hospitalized Older Adults by Year of Hospital Admission

Characteristic	Older adults admitted to acute care hospitals, No. (%)						Standardized difference^b^
	2017 (n = 407 999)	2018 (n = 417 851)	2019 (n = 424 606)	2020 (n = 376 761)	2021 (n = 401 813)	2022 (n = 99 381)^a^	
Age, mean (SD), y	78.9 (8.3)	78.9 (8.3)	78.9 (8.3)	78.9 (8.3)	78.9 (8.3)	79.2 (8.3)	0.004
Age category, y							
66-74	145 103 (35.6)	148 893 (35.6)	152 839 (36.0)	135 715 (36.0)	143 722 (35.8)	33 849 (34.1)	0.001
75-84	150 652 (36.9)	153 835 (36.8)	155 347 (36.6)	138 263 (36.7)	148 485 (37.0)	37 078 (37.3)	0.003
≥85	112 244 (27.5)	115 123 (27.6)	116 420 (27.4)	102 783 (27.3)	109 606 (27.3)	28 454 (28.6)	0.002
Sex							
Female	208 622 (51.1)	213 492 (51.1)	215 117 (50.7)	188 834 (50.1)	202 693 (50.4)	49 552 (49.9)	0.016
Male	199 377 (48.9)	204 359 (48.9)	209 289 (49.3)	187 927 (49.9)	199 120 (49.6)	49 829 (50.1)	0.016
Chronic conditions, No.							
0	42 075 (10.3)	44 403 (10.6)	46 426 (10.9)	43 437 (11.5)	54 231 (13.5)	15 266 (15.4)	0.074
1	97 909 (24.0)	103 211 (24.7)	106 632 (25.1)	99 582 (26.4)	115 028 (28.6)	30 103 (30.3)	0.077
2	110 864 (27.2)	115 710 (27.7)	118 557 (27.9)	106 258 (28.2)	113 412 (28.2)	27 435 (27.6)	0.012
3	82 933 (20.3)	83 921 (20.1)	84 875 (20.0)	72 429 (19.2)	70 760 (17.6)	16 306 (16.4)	0.054
4	45 328 (11.1)	44 184 (10.6)	43 826 (10.3)	36 153 (9.6)	32 542 (8.1)	7097 (7.1)	0.072
≥5	28 890 (7.1)	26 422 (6.3)	24 290 (5.7)	18 902 (5.0)	15 840 (3.9)	3174 (3.2)	0.094
Dementia	29 088 (7.1)	25 981 (6.2)	22 491 (5.3)	18 574 (4.9)	18 749 (4.7)	4784 (4.8)	0.060
Income quintile^c^							
1	98 669 (24.2)	100 969 (24.2)	101 588 (24.0)	89 404 (23.7)	94 568 (23.5)	23 819 (24.0)	0.011
2	89 230 (21.9)	91 173 (21.8)	91 840 (21.6)	81 359 (21.6)	86 068 (21.6)	21 184 (21.3)	0.007
3	79 269 (19.4)	80 559 (19.3)	82 321 (19.4)	73 100 (19.4)	77 936 (19.4)	19 085 (19.2)	0.000
4	70 895 (17.4)	72 992 (17.5)	74 800 (17.6)	66 602 (17.7)	71 755 (17.9)	17 751 (17.9)	0.008
5	69 936 (17.1)	72 158 (17.3)	74 057 (17.4)	66 296 (17.6)	71 486 (17.8)	17 542 (17.7)	0.011
ICU admission	50 503 (12.4)	51 507 (12.3)	52 103 (12.3)	46 804 (12.4)	47 340 (11.8)	11 813 (11.9)	0.007
Nursing home resident	27 060 (6.6)	27 230 (6.5)	26 508 (6.2)	18 940 (5.0)	16 840 (4.2)	4501 (4.5)	0.088
Rural resident^d^	56 599 (13.9)	56 371 (13.5)	56 778 (13.4)	50 137 (13.3)	53 197 (13.2)	12 829 (12.9)	0.009
Immigrant to Canada	28 677 (7.0)	30 995 (7.4)	33 563 (7.9)	29 984 (8.0)	34 886 (8.7)	9330 (9.4)	0.037

Abbreviation: ICU, intensive care unit.

^a^ Included 3 months, from January 1 to March 31, 2022.

^b^ Standardized difference between the prepandemic and pandemic periods. A threshold of 0.100 was selected a priori to represent a meaningful difference. Prepandemic included all months from January 1, 2017, to February 29, 2020, and postpandemic included March 1, 2020, to March 31, 2022.

^c^ Data were missing for 8129 individuals (0.4%).

^d^ Data were missing for 7303 individuals (0.3%).

**Figure 1.**
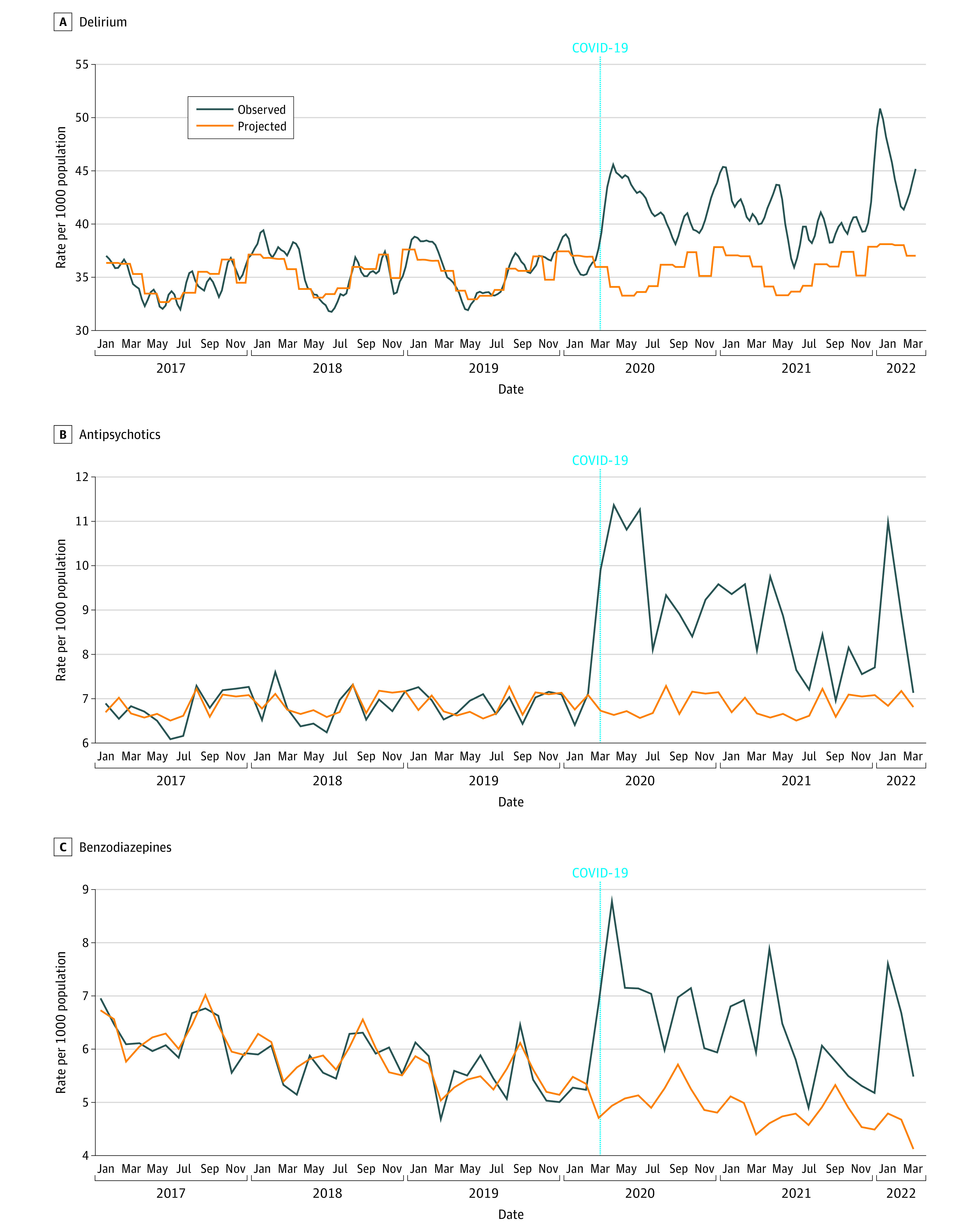
Rates of Delirium and Use of New Antipsychotics and Benzodiazepines Before and After the Onset of the COVID-19 Pandemic

Rates of delirium increased overall from 35.9 per 1000 admitted population before the pandemic to 41.5 per 1000 admitted population during the pandemic. Monthly rates of new prescriptions for antipsychotics increased from 6.9 to 8.8 per 1000 discharged population, and monthly rates of new benzodiazepine prescriptions increased from 4.4 to 6.0 per 1000 discharged population. The rates of delirium and medications by month and year are shown in Figure 1 and eTables 5-7 in Supplement 1. During the pandemic, adjusted rates of delirium were higher compared with projected rates (ARR, 1.15; 95% CI, 1.11-1.19). Rates of new prescriptions for antipsychotics were higher (ARR, 1.28; 95% CI, 1.19-1.38) compared with projected rates, as were rates of new benzodiazepine prescriptions (ARR, 1.37; 95% CI, 1.20-1.57). The largest monthly increases for all outcomes occurred during pandemic waves 1, 3, and 5. Although rates were relatively lower in other months, they were elevated above projected levels throughout the pandemic even when provincial cases of COVID-19 were low.

In sensitivity analyses, of 806 555 hospital admissions after the onset of the COVID-19 pandemic, there were 42 690 admissions with COVID-19 (5.3%), and excluding them did not change the findings (delirium rate: 35.6 vs 40.3 per 1000 admitted population; ARR, 1.12; 95% CI, 1.09-1.16). Likewise, excluding admissions with a diagnosis of COVID-19 did not change rates of newly dispensed antipsychotics (ARR, 1.28; 95% CI, 1.18-1.30) or benzodiazepines (ARR, 1.37; 95% CI, 1.19-1.57).

**Figure 2.**
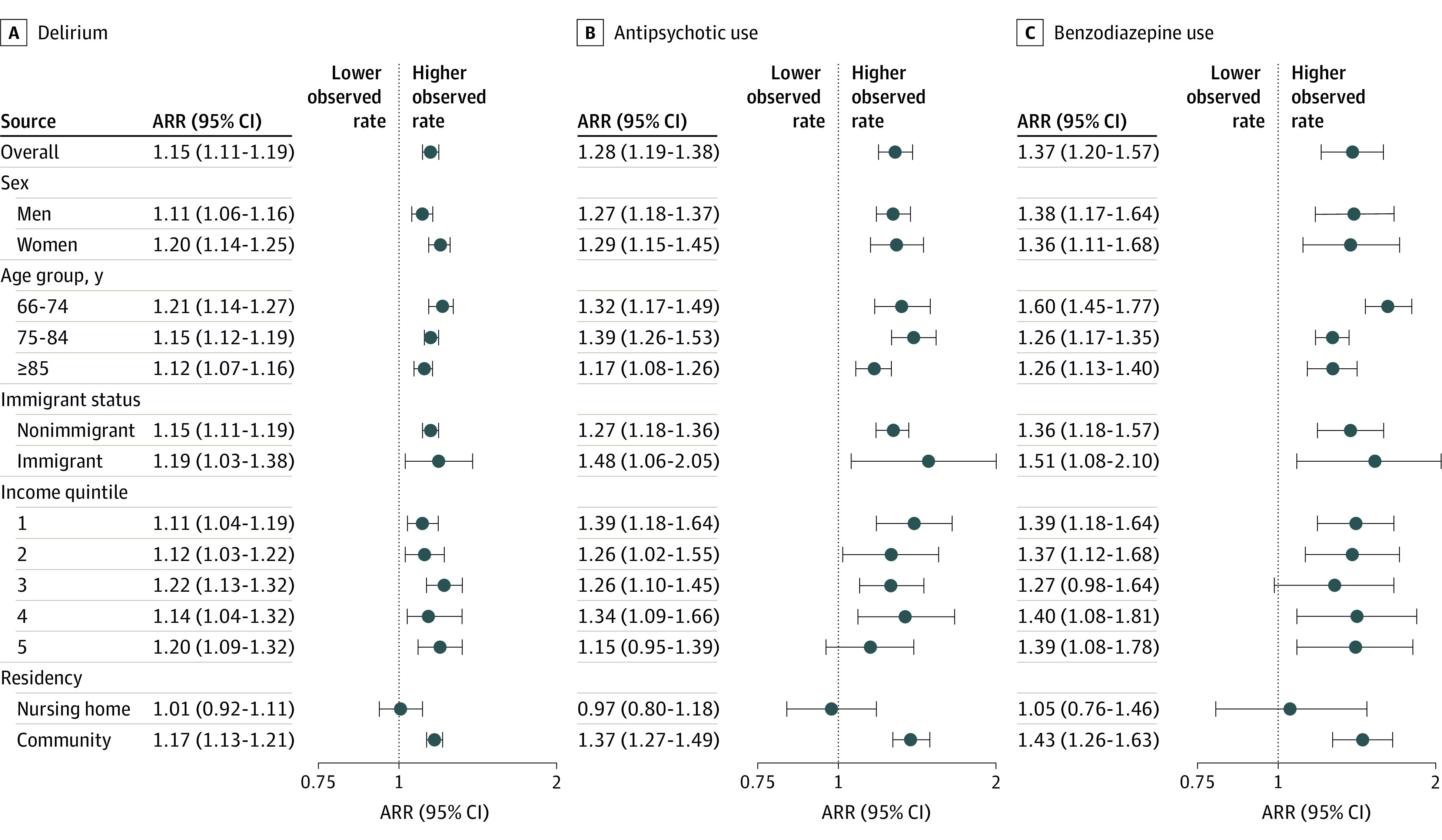
Adjusted Rate Ratios (ARRs) for Delirium and Use of Antipsychotics and Benzodiazepines Overall and by Sex, Age Group, Immigrant Status, Income, and Place of Residence Markers represent ARRs, with horizontal lines representing 95% CIs.

We found a gradient in increased delirium rates by age group, with younger age groups experiencing higher delirium rates compared with projected trends, but the difference was not significant (Figure 2). Delirium rates were higher than projected rates for both men and women, with higher rates observed for women (ARR, 1.20; 95% CI, 1.14-1.25). Both immigrants and nonimmigrants as well as older adults from different income quintiles experienced similar increases in delirium rates during the pandemic. Community-dwelling older adults experienced an increase in delirium rates (ARR, 1.17; 95% CI, 1.13-1.21), although nursing home residents did not. All age groups experienced higher than projected rates of new antipsychotic and benzodiazepine prescriptions at hospital discharge. The increase was most pronounced for new benzodiazepines in those aged 66 to 74 years (ARR, 1.60; 95% CI, 1.45-1.77). Rates of prescriptions for new antipsychotics and benzodiazepines for nursing home residents did not change, while community-dwelling older adults had rates that were above projected rates for new antipsychotics (ARR, 1.37; 95% CI, 1.27-1.49) and new benzodiazepines (ARR, 1.43; 95% CI, 1.26-1.63). Complete tabulation for observed and projected outcomes overall and within subgroups is available in eTable 8 in Supplement 1.

## Discussion

In this population-based study of hospitalized older adults in Ontario, there were significant increases in rates of delirium and new antipsychotic and benzodiazepine prescriptions after the onset of the COVID-19 pandemic. Rates of delirium and related medication prescriptions were elevated above projected rates even when provincial cases of COVID-19 were low, suggesting that there were other pandemic-related factors involved beyond COVID-19. This finding was supported by our sensitivity analysis, in which removing COVID-19–related hospital admissions did not change the findings.

Pandemic-related disruptions have been well documented in the nursing home setting, with several studies highlighting increased antipsychotic prescribing rates.^27,28,29^ Similar to our study, discerning factors associated with increased prescribing is difficult but may reflect a lack of staffing, visitor restrictions, and reduced opportunity for patients with dementia to benefit from nonpharmacologic interventions due to in-room isolation.^30^ The COVID-19 pandemic created similar conditions for delirium in hospital. A recent study of patients admitted with stroke showed that pandemic-associated visitation restrictions were associated with a higher incidence of delirium.^31^ Mandated infection control procedures also led to prolonged periods during which older patients were in bed and isolated, and the use of masks and face shields could have disrupted communication during patient and staff interactions.^32^ Staff shortages limited opportunities to implement evidenced-based strategies for delirium, including assistance with feeding to support nutrition, mobility out of bed, and frequent orientation.^4^ These disruptions may have been associated with increased risk of delirium and increased risk of pharmacologic treatment being used over nonpharmacologic strategies. Since we could not ascertain inpatient medication use, we believe that these drugs were more frequently used or there were pandemic-related disruptions to medication reconciliation that were associated with increased risk of medications being continued after discharge.^33^ Regardless, our finding that these medications were continued after discharge is concerning, as long-term use of these medications in older adults is associated with adverse events, such as falls and stroke.^34^

Even though exclusion of COVID-19 diagnoses in a sensitivity analysis did not affect our results, it is plausible that COVID-19 contributed in part to the observed associations. Delirium is known to occur in over half of older adults hospitalized with SARS-CoV-2 infection,^35^ and our measure of COVID-19 with *ICD-10* codes (5.3%) may have undercounted cases compared with laboratory testing, which indicated that hospitalizations due to confirmed COVID-19 were closer to 8% over a similar period.^36^ It is possible that antipsychotic and benzodiazepine use also increased to treat agitation resulting from COVID-19–related delirium. This notion is supported by a study that found that patients admitted with COVID-19 required higher doses of antipsychotics to treat delirium symptoms compared with patients who had delirium from other conditions.^37^ Since benzodiazepines are used less frequently for delirium, another mechanism for the observed increase during the pandemic could be related to treatment for anxiety or sleep disturbance, which was known to be highly prevalent among hospitalized patients with COVID-19.^38^ The increase in benzodiazepine use is particularly worrisome since our data showed that the rates of prescriptions after discharge were decreasing over time between 2017 and 2020 and this progress was disrupted by the pandemic.

The long-standing consequence of increased delirium during the pandemic is particularly concerning. Delirium has an association with future dementia, not just as a marker of cognitive vulnerability but as a contributor to permanent neuronal damage.^39^ Cognitive decline after COVID-19 has been documented on a population level up to a year after infection.^40,41^ Whether delirium acts as a mediator in the association between COVID-19 and long-term cognitive consequences should be an area of future study. We found that the largest increases in delirium were in the youngest age group studied (66-74 years), which was unexpected given that delirium risk increases with age. The potential for prolonged cognitive impairment in this group may have important personal and societal repercussions, including early retirement and/or earlier development of dementia. Women experienced a greater relative increase in delirium rates than men, which may compound future cognitive risk for older women, who are already disproportionately affected by Alzheimer disease.^42^ These findings are relavant to cognitive rehabilitation initiatives for the post–COVID-19 condition, which could consider targeting older patients who experienced delirium and preparing to serve more women and adults younger than 75 years.^43^

### Limitations

This study has several limitations. As with all observational studies, there was potential for residual confounding, although the rapid rate increases that coincided with pandemic onset are unlikely to be explained by some other contemporaneous factor. The finding of the association with the pandemic was also strengthened by the long duration of prepandemic data we used to estimate future trends. Delirium rates might be underestimated if people admitted after the pandemic were at higher risk for delirium. This is unlikely, as mean age was the same by year of admission and the comorbidity burden of the admitted population marginally decreased during the pandemic. Misclassification of delirium from *ICD-10* codes is expected because the codes are insensitive, although there is no reason to believe that misclassification occurred differentially before and after the pandemic. Additionally, the specificity of diagnostic codes for delirium is high, so our findings of increased delirium rates are likely accurate although underrepresentative of true delirium incidence. It is also possible that delirium coding improved over time, yet there were no targeted initiatives or widespread coding changes that coincided with the onset of the pandemic. It is likely that antipsychotic and benzodiazepine use was higher than reported since we could not ascertain inpatient medications and we could only measure dispensed prescriptions for patients who were discharged, excluding those who died and may have had critical illness or terminal delirium. We only considered antipsychotics commonly used for delirium and excluded patients with schizophrenia, although it is possible that antipsychotics were newly prescribed for behavioral symptoms of dementia or a new diagnosis of schizophrenia.

## Conclusions

In this cross-sectional study, there was a temporal association between COVID-19 pandemic onset and increases in rates of in-hospital delirium and new antipsychotic and benzodiazepine prescriptions after hospital discharge among older adults. Rates recovered from their peak but remained elevated above projected levels throughout the first 2 years of the pandemic. Overall, these findings suggest that pandemic-related disruptions to care were associated with the observed increases in delirium and related medication prescribing. Increases in delirium and continuation of antipsychotic and benzodiazepine medications may have lasting consequences for the older population, including increased burden of cognitive impairment, functional decline, and medication-related adverse effects. Health systems and clinicians should renew their efforts to implement well-described nonpharmacologic interventions to prevent and manage delirium in the hospital. Government and hospital policies are imminently needed to address ongoing staff shortages, mandate flexible hospital visitation, and consider delirium care in future decision-making about isolation practices.

## Notes

Supplement 1. **eAppendix.** Methodologic Details Regarding Administrative Data Sets **eTable 1.** Description of Data Sources **eTable 2.** Data Sources for 17 Chronic Conditions **eTable 3.** *ICD-10* and Physician Claims Fee Codes Used to Identify Variables **eTable 4.** List of Study Medications From ODB Database **eFigure.** Cohort Creation Flow Chart **eTable 5.** Observed and Expected Rates and Adjusted Rate Ratios of Delirium by Month and Pandemic Wave **eTable 6.** Observed and Expected Rates and Adjusted Rate Ratios of New Antipsychotic Prescriptions by Month and Pandemic Wave **eTable 7.** Observed and Expected Rates and Adjusted Rate Ratios of New Benzodiazepine Prescriptions by Month and Pandemic Wave **eTable 8.** Observed and Expected Rates and Adjusted Rate Ratios for Delirium, Antipsychotics, and Benzodiazepines Overall and by Subgroups
eReferences

Supplement 2.
Data Sharing Statement
