# Supplementary material for: Trends in Delirium and New Antipsychotic and Benzodiazepine Use Among Hospitalized Older Adults Before and After the Onset of the COVID-19 Pandemic
Source: JAMA Netw Open. 2023 Aug 7;6(8):e2327750. doi: 10.1001/jamanetworkopen.2023.27750 (PMC10407689; doi:10.1001/jamanetworkopen.2023.27750)
Supplement: Supplement 1. — eAppendix. Methodologic Details Regarding Administrative Data Sets eTable 1. Description of Data Sources eTable 2. Data Sources for 17 Chronic Conditions eTable 3. ICD-10 and Physician Claims Fee Codes Used to Identify Variables eTable 4. List of Study Medications From ODB Database eFigure. Cohort Creation Flow Chart eTable 5. Observed and Expected Rates and Adjusted Rate Ratios of Delirium by Month and Pandemic Wave eTable 6. Observed and Expected Rates and Adjusted Rate Ratios of New Antipsychotic Prescriptions by Month and Pandemic Wave eTable 7. Observed and Expected Rates and Adjusted Rate Ratios of New Benzodiazepine Prescriptions by Month and Pandemic Wave eTable 8. Observed and Expected Rates and Adjusted Rate Ratios for Delirium, Antipsychotics, and Benzodiazepines Overall and by Subgroups eReferences [file jamanetwopen-e2327750-s001.pdf]

Supplementary Online Content

Reppas-Rindlisbacher C, Boblitz A, Fowler RA, et al. Trends in delirium and new antipsychotic and benzodiazepine use among hospitalized older adults before and after the onset of the COVID-19 pandemic. *JAMA Netw Open*. 2023;6(8):e2327750.
doi:10.1001/jamanetworkopen.2023.27750

eAppendix. Methodologic Details Regarding Administrative Data Sets
eTable 1. Description of Data Sources
eTable 2. Data Sources for 17 Chronic Conditions
eTable 3. *ICD-10* and Physician Claims Fee Codes Used to Identify Variables
eTable 4. List of Study Medications From ODB Database
eFigure. Cohort Creation Flow Chart
eTable 5. Observed and Expected Rates and Adjusted Rate Ratios of Delirium by Month and Pandemic Wave
eTable 6. Observed and Expected Rates and Adjusted Rate Ratios of New Antipsychotic Prescriptions by Month and Pandemic Wave
eTable 7. Observed and Expected Rates and Adjusted Rate Ratios of New Benzodiazepine Prescriptions by Month and Pandemic Wave
eTable 8. Observed and Expected Rates and Adjusted Rate Ratios for Delirium, Antipsychotics, and Benzodiazepines Overall and by Subgroups
eReferences

This supplementary material has been provided by the authors to give readers additional information about their work.

© 2023 Reppas-Rindlisbacher C et al. *JAMA Network Open*.

eAppendix: Methodologic details regarding administrative data sets

All residents of Ontario have universal access to hospital care, physicians' services, and those aged ≥ 65 years of age are provided universal prescription drug insurance coverage without the requirement for co-payment. The administrative datasets used in this study were linked using encoded identifiers at the patient level and analyzed at ICES. For all datasets, cleaning and data preparation were undertaken. Individuals with missing age or sex were excluded from the study entirely. Implausible values from RPDB and CIC-IRCC (income quintile, rural resident, immigrant to Canada) were set to missing. Information on hospital admissions and diagnoses are coded by trained personnel using the International Classification of Diseases, 10th Revision (ICD-10) system; personnel only consider physician-recorded diagnoses in a patient's medical chart when assigning codes and do not review or interpret symptoms or test results.

© 2023 Reppas-Rindlisbacher C et al. *JAMA Network Open*.

eTable 1. Description of data sources

Database	Description
Canadian Institute for Health Information Discharge Abstract Database (CIHI-DAD)	Contains detailed diagnostic and procedural information for all hospital admissions in Canada. DAD records have been demonstrated to have excellent agreement (over 99%) for demographic and administrative data. Regarding diagnoses, median agreement between original DAD records and re-abstracted records for the 50 most common most responsible diagnoses was noted to be 81% (Sensitivity 82%; Specificity 82%). The corresponding median agreement for the 50 most frequently performed surgical procedures was 92% (sensitivity 95%, positive predictive value 91%).[1]
Registered Persons Database (RPDB)	Registry of detailed demographic information for all Ontarians eligible to receive insured health services in the province.
Postal Code Conversion File (PCCF)	The Postal Code Conversion File (PCCF) links six-character postal codes to standard geographic areas such as dissemination areas, census tracts, and census subdivisions.
Continuing Care Reporting System Long-Term Care (CCRS-LTC)	Contains demographic, administrative, clinical and resource utilization information on patients who receive continuing care services in hospitals or long term care (LTC) homes in Canada.
Ontario Drug Benefit (ODB)	Provides individual prescription records including all prescriptions dispensed to Ontario residents aged 65 years and older. Each medication claim has an associated prescriber identifier which indicates the health practitioner who wrote the prescription. An audit of 5,155 randomly selected prescriptions dispensed from 50 Ontario pharmacies determined that the ODB had an error rate of 0.7% and none of the pharmacy characteristics examined (locations, owner affiliation, productivity) were associated with coding errors.[2]
Ontario Health Insurance Plan (OHIP)	Identifies physician billing claims and specialty on all services provided by fee-for-service physicians in Ontario.
Immigration, Refugees and Citizenship Canada (IRCC)	Includes demographic information for all immigrants to Canada from January 1, 1985 to September 30, 2020. Linkage of Immigration, Refugees and Citizenship Canada data to population registries has been validated with a linkage rate of 86% in Ontario.[3]
National Ambulatory Care Reporting System (NACRS)	Reports demographic, administrative, clinical and service-specific data for Emergency Department visits
Same Day Surgery (SDS)	Contains patient-level data for day surgery institutions in Ontario. Every record corresponds to one same-day surgery or procedure stay
Ontario Mental Health Reporting System (OMHRS)	Documents data on patients in adult designated inpatient mental health beds. This includes beds in General, Provincial Psychiatric, and Specialty Psychiatric facilities.

© 2023 Reppas-Rindlisbacher C et al. *JAMA Network Open*.

eTable 2. Data Sources for 17 Chronic Conditions

Condition	Existing Cohort or Algorithm (Look-back Window)	Source
Arthritis: At least one of rheumatoid arthritis[5,6]; osteoarthritis	Rheumatoid: Existing cohort; Osteoarthritis: Algorithm (10 years)	CIHI-DAD, OHIP, RPDB
Asthma[7,8,9]	Existing cohort	CIHI-DAD, OHIP, CIHI-SDS, RPDB
Cancer	Algorithm (5 years)	CIHI-DAD, OHIP, RPDB
CHF[10]	Existing cohort	CIHI-DAD, OHIP, NACRS, OMHRS, RPDB
COPD including bronchitis[11]	Existing cohort	CIHI-DAD, OHIP, CIHI-SDS, RPDB
CVD: 1+ coronary artery disease including MI, angina; peripheral vascular disease; arrhythmia[12]	Algorithm (5 years)	CIHI-DAD, OHIP, RPDB
Dementia	Algorithm (10 years)	CIHI-DAD, OHIP, ODB, RPDB
Diabetes[13,14]	Existing cohort	CIHI-DAD, OHIP CIHI-SDS, NACRS, RPDB
HIV[15]	Existing cohort	OHIP, RPDB
Hypertension[16,17]	Existing cohort	CIHI-DAD, OHIP, RPDB
IBD[18,19]	Existing cohort	CIHI-DAD, OHIP, NACRS, ODB, RPDB
Kidney dx, chronic	Algorithm (10 years)	CIHI-DAD, OHIP, RPDB
Liver disease, chronic	Algorithm (10 years)	CIHI-DAD, OHIP, RPDB
Mood disorder: 1+ depression; anxiety; phobia; bipolar disorder	Algorithm (5 years)	CIHI-DAD, OHIP, OMHRS, RPDB
Osteoporosis	Algorithm (10 years)	CIHI-DAD, OHIP, RPDB
Stoke / TIA	Algorithm (10 years)	CIHI-DAD, OHIP, RPDB
Urinary incontinence	Algorithm (5 years)	CIHI-DAD, OHIP, RPDB

© 2023 Reppas-Rindlisbacher C et al. *JAMA Network Open*.

eTable 3. *ICD-10* and Physician claims fee codes used to identify variables

Delirium	CIHI-DAD	ICD-10:<br>• F050 Delirium not superimposed on dementia<br>• F051 Delirium superimposed on dementia<br>• F058 Other delirium<br>• F059 Delirium unspecified
COVID-19 Infection	CIHI DAD	ICD-10*:<br>• U07.1 (diagnosis of COVID-19 confirmed by laboratory testing)<br>• U07.2 (clinical or epidemiological diagnosis of COVID-19 where laboratory confirmation is inconclusive or not available)
	OHIP	OHIP dx: 080
Schizophrenia (for exclusions)	CIHI-DAD<br>OMHRS	ICD10 codes: F20 ,F25<br>ICD9 codes: 29381, 29382, 295, 297, 298
	OHIP	OHIP dx: 295

* In 15 US hospitals these codes were 98% sensitive and 99% specific for COVID-19[4]

© 2023 Reppas-Rindlisbacher C et al. *JAMA Network Open*.

eTable 4. List of Study Medications (from ODB database)

Antipsychotics (for lookback to define new)	amisulpride, aripiprazole, asenapine, chlorpromazine, chlorprothixene, clozapine, flupentixol, fluphenazine, haloperidol, loxapine, lurasidone, mesoridazine, methotrimeprazine, olanzapine, paliperidone, periciazine, perphenazine, pimozide, quetiapine, risperidone, thiopropazate, thioridazine, trifluoperazine, ziprasidone, zuclopenthixol
Antipsychotics (for study outcome)	Risperidone, quetiapine, olanzapine, haloperidol, loxapine, aripiprazole
Benzodiazepines* (for lookback and study outcome)	alprazolam, bromazepam, chlordiazepoxide, clonazepam, clorazepate, diazepam, flurazepam, ketazolam, lorazepam, nitrazepam, oxazepam, temazepam, triazolam

*did not include zopiclone or zolpidem

© 2023 Reppas-Rindlisbacher C et al. *JAMA Network Open*.

eFigure. Cohort Creation Flow Chart

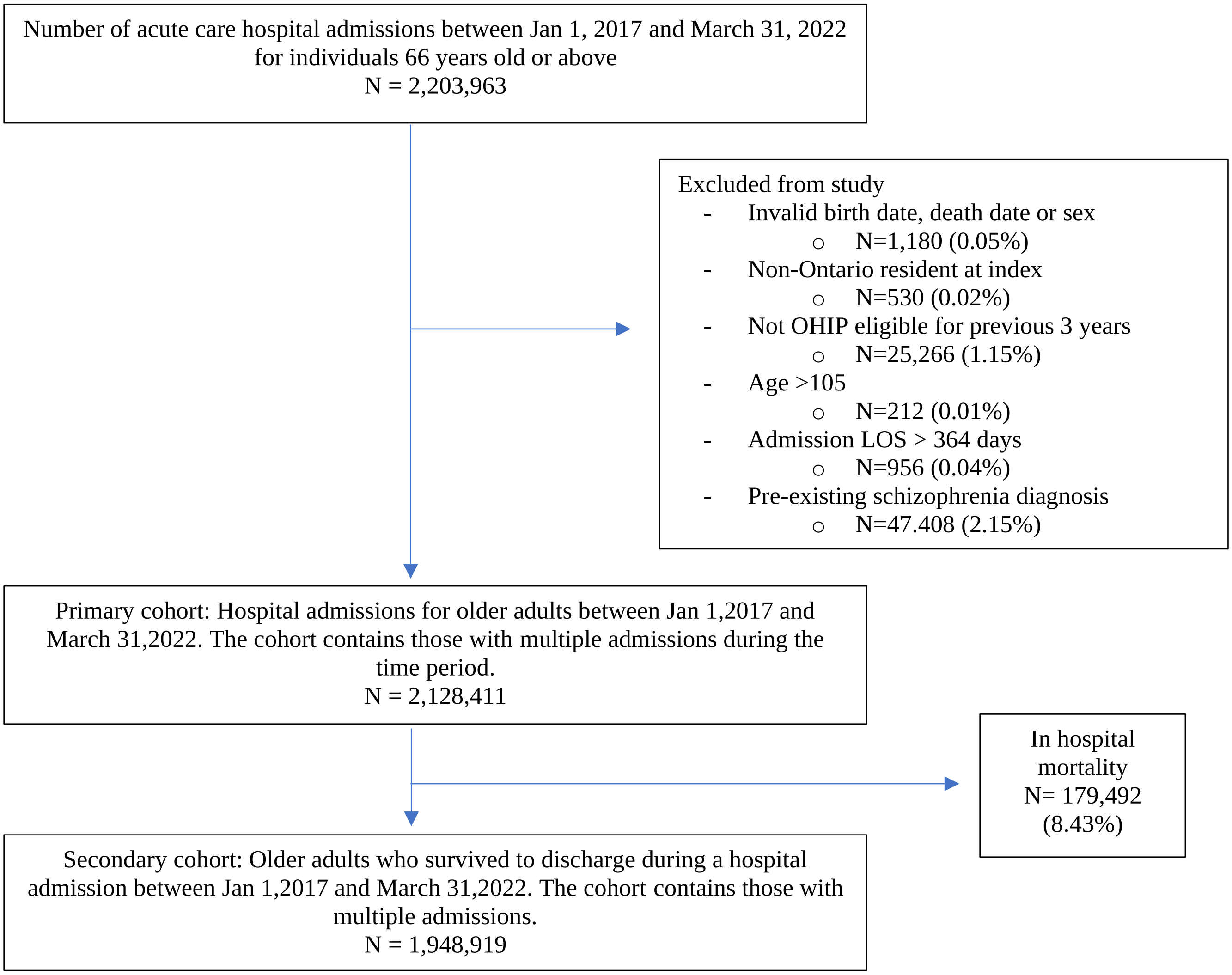

© 2023 Reppas-Rindlisbacher C et al. *JAMA Network Open*.

eTable 5. Observed and expected rates and adjusted rate ratios (aRR, 95% CI) of delirium by month and pandemic wave

Pandemic Wave	Year and Month	Observed Rate	Expected Rate	Monthly aRR (95% CI)	Pandemic wave aRR (95% CI)
	Overall	41.4	35.9	1.15 (1.11, 1.19)	
	**2020**				
1	March	38.3	36.0	1.07 (1.03, 1.10)	1.24 (1.20, 1.28)
	April	45.2	34.1	1.33 (1.29, 1.36)	
	May	44.3	33.3	1.33 (1.30, 1.37)	
	June	43.2	33.6	1.29 (1.24, 1.34)	
	July	41.1	34.2	1.20 (1.16, 1.24)	
	August	40.1	36.2	1.11 (1.07, 1.15)	
2	September	39.9	36.0	1.11 (1.05, 1.17)	1.13 (1.09, 1.17)
	October	39.5	37.4	1.06 (1.02, 1.09)	
	November	40.8	35.2	1.16 (1.13, 1.20)	
	December	44.4	37.9	1.17 (1.15, 1.19)	
	**2021**				
	January	42.9	37.1	1.16 (1.12, 1.20)	
	February	41.5	37.0	1.12 (1.10, 1.14)	
3	March	39.9	36.0	1.11 (1.07, 1.15)	1.17 (1.13, 1.21)
	April	42.0	34.1	1.23 (1.20, 1.27)	
	May	41.9	33.3	1.26 (1.22, 1.29)	
	June	36.8	33.6	1.09 (1.05, 1.14)	
	July	39.0	34.2	1.14 (1.10, 1.18)	
	August	40.3	36.2	1.11 (1.07, 1.15)	
4	September	39.0	36.0	1.08 (1.02, 1.15)	1.10 (1.05, 1.14)
	October	40.0	37.4	1.07 (1.04, 1.11)	
	November	39.9	35.2	1.13 (1.09, 1.17)	
5	December	44.0	37.9	1.16 (1.15, 1.18)	1.19 (1.16, 1.22)
	**2022**				
	January	48.4	38.1414	1.27 (1.22, 1.32)	
	February	42.7	38.055	1.12 (1.10, 1.15)	
	March	43.2	37.0	1.17 (1.12, 1.21)	

*adjusted for age-group, sex, linear term of weeks since January 1, 2017, and pre-COVID-19 month indicators to model seasonal variations

© 2023 Reppas-Rindlisbacher C et al. *JAMA Network Open*.

eTable 6. Observed and expected rates and adjusted rate ratios (aRR, 95% CI) of new antipsychotic prescriptions by month and pandemic wave

Pandemic Wave	Year and Month	Observed Rate	Expected Rate	Monthly aRR (95% CI)*	Pandemic wave aRR (95% CI)
	Overall	8.8	6.9	1.28 (1.19, 1.38)	
	**2020**				
1	March	9.9	6.7	1.47 (1.39, 1.56)	1.62 (1.51, 1.75)
	April	11.3	6.6	1.72 (1.59, 1.85)	
	May	10.8	6.7	1.61 (1.45, 1.79)	
	June	11.3	6.5	1.72 (1.62, 1.83)	
	July	8.1	6.7	1.22 (1.14, 1.30)	
	August	9.3	7.3	1.28 (1.21, 1.36)	
2	September	8.9	6.6	1.34 (1.24, 1.45)	1.29 (1.19, 1.41)
	October	8.4	7.1	1.17 (1.08, 1.27)	
	November	9.2	7.1	1.30 (1.25, 1.35)	
	December	9.6	7.1	1.34 (1.28, 1.41)	
	**2021**				
	January	9.3	6.7	1.40 (1.28, 1.52)	
	February	9.6	7.0	1.37 (1.27, 1.47)	
3	March	8.1	6.6	1.21 (1.13, 1.30)	1.32 (1.23, 1.41)
	April	9.7	6.5	1.49 (1.37, 1.62)	
	May	8.9	6.6	1.33 (1.19, 1.50)	
	June	7.6	6.5	1.18 (1.09, 1.27)	
	July	7.2	6.6	1.09 (1.02, 1.16)	
	August	8.4	7.2	1.17 (1.10, 1.25)	
4	September	6.9	6.6	1.06 (0.96, 1.16)	1.09 (1.01, 1.19)
	October	8.1	7.1	1.15 (1.05, 1.26)	
	November	7.5	7.0	1.07 (1.02, 1.13)	
5	December	7.7	7.1	1.09 (1.03, 1.15)	1.30 (1.20, 1.42)
	**2022**				
	January	11.0	6.8	1.61 (1.46, 1.78)	
	February	8.9	7.2	1.24 (1.14, 1.35)	
	March	7.1	6.8	1.05 (0.96, 1.14)	

*adjusted for age-group, sex, linear term of weeks since January 1, 2017, and pre-COVID-19 month indicators to model seasonal variations

© 2023 Reppas-Rindlisbacher C et al. *JAMA Network Open*.

eTable 7. Observed and expected rates and adjusted rate ratios (aRR, 95% CI) of new benzodiazepine prescriptions by month and pandemic wave

Pandemic Wave	Year and Month	Observed Rate	Expected Rate	Monthly aRR (95% CI)*	Pandemic wave aRR (95% CI)
	Overall	6.0	4.4	1.37 (1.20, 1.57)	
	**2020**				
1	March	6.5	4.3	1.50 (1.36, 1.66)	1.54 (1.35, 1.75)
	April	8.4	4.5	1.85 (1.58, 2.18)	
	May	6.8	4.7	1.45 (1.25, 1.67)	
	June	6.8	4.7	1.43 (1.28, 1.60)	
	July	6.7	4.5	1.48 (1.44, 1.52)	
	August	5.6	4.9	1.15 (1.04, 1.27)	
2	September	6.6	5.3	1.24 (1.09, 1.41)	1.32 (1.18, 1.48)
	October	6.8	4.9	1.39 (1.23, 1.58)	
	November	5.6	4.5	1.26 (1.12, 1.42)	
	December	5.5	4.4	1.26 (1.13, 1.41)	
	**2021**				
	January	6.4	4.7	1.36 (1.21, 1.54)	
	February	6.5	4.6	1.42 (1.30, 1.56)	
3	March	5.5	4.0	1.39 (1.21, 1.59)	1.44 (1.22, 1.70)
	April	7.5	4.2	1.78 (1.46, 2.19)	
	May	6.1	4.3	1.41 (1.17, 1.69)	
	June	5.4	4.4	1.23 (1.06, 1.43)	
	July	4.5	4.2	1.08 (1.01, 1.15)	
	August	5.7	4.5	1.26 (1.10, 1.44)	
4	September	5.4	4.9	1.09 (0.93, 1.28)	1.13 (0.97, 1.33)
	October	5.1	4.5	1.13 (0.96, 1.33)	
	November	4.9	4.1	1.19 (1.02, 1.39)	
5	December	4.8	4.1	1.17 (1.02, 1.35)	1.42 (1.24, 1.64)
	**2022**				
	January	7.2	4.4	1.65 (1.41, 1.93)	
	February	6.3	4.3	1.47 (1.30, 1.66)	
	March	5.1	3.7	1.37 (1.15, 1.63)	

*adjusted for age-group, sex, linear term of weeks since January 1, 2017, and pre-COVID-19 month indicators to model seasonal variations

© 2023 Reppas-Rindlisbacher C et al. *JAMA Network Open*.

eTable 8. Observed and Expected rates and adjusted rate ratios (aRR, 95%CI) for Delirium, Antipsychotics and Benzodiazepines Overall and by Subgroups

	Delirium			Antipsychotics			Benzodiazepines		
	Observed	Expected	aRR (95%CI)	Observed	Expected	aRR (95%CI)	Observed	Expected	aRR (95%CI)
**Overall**	41.4	35.9	1.15 (1.11-1.19)	8.8	6.9	1.28 (1.19-1.38)	6.0	4.4	1.37 (1.20-1.57)
**Men**	40.0	36.0	1.11 (1.06-1.16)	8.5	6.7	1.27 (1.18-1.37)	6.1	4.4	1.38 (1.17-1.64)
**Women**	42.9	35.9	1.20 (1.14-1.25)	9.1	7.0	1.29 (1.15-1.45)	5.9	4.3	1.36 (1.11-1.68)
**66-74**	22.3	18.5	1.21 (1.14-1.27)	5.4	4.1	1.32 (1.17-1.49)	5.5	3.4	1.60 (1.45-1.77)
**75-84**	41.1	35.6	1.15 (1.12-1.19)	8.5	6.1	1.39 (1.26-1.53)	5.7	4.5	1.26 (1.17-1.35)
**85+**	66.9	59.9	1.12 (1.07-1.16)	14.1	12.0	1.17 (1.08-1.26)	7.0	5.6	1.26 (1.13-1.40)
**Non-Immigrant**	41.9	36.4	1.15 (1.11-1.19)	8.7	6.9	1.27 (1.18-1.36)	5.9	4.3	1.36 (1.18-1.57)
**Immigrant**	36.7	30.8	1.19 (1.03-1.38)	9.5	6.4	1.48 (1.06-2.05)	6.9	4.6	1.51 (1.08-2.10)
**Income Quintile**									
**1 (lowest)**	43.8	39.4	1.11 (1.04-1.19)	8.9	6.4	1.39 (1.18-1.64)	5.7	3.9	1.39 (1.18-1.64)
**2**	42.4	37.8	1.12 (1.03-1.22)	8.8	7.0	1.26 (1.02-1.55)	5.8	4.2	1.37 (1.12-1.68)
**3**	41.6	34.0	1.22 (1.13-1.32)	8.9	7.0	1.26 (1.10-1.45)	6.1	4.8	1.27 (0.98-1.64)
**4**	39.2	34.4	1.14 (1.04-1.32)	9.0	6.7	1.34 (1.09-1.66)	6.2	4.4	1.40 (1.08-1.81)
**5 (highest)**	39.7	32.8	1.20 (1.09-1.32)	8.4	7.4	1.15 (0.95-1.39)	6.2	4.5	1.39 (1.08-1.78)
**Nursing Home**	55.6	55.1	1.01 (0.92-1.11)	21.8	22.5	0.97 (0.80-1.18)	10.9	10.3	1.05 (0.76-1.46)
**Community**	40.8	34.9	1.17 (1.13-1.21)	8.3	6.0	1.37 (1.27-1.49)	5.8	4.0	1.43 (1.26-1.63)

© 2023 Reppas-Rindlisbacher C et al. *JAMA Network Open*.

eReferences

1. Juurlink DN, Preyra C, Croxford R, et al. Canadian Institute for Health Information Discharge Abstract Database: A Validation Study.; 2006.
2. Levy AR, O'Brien BJ, Sellors C, Grootendorst P, Willison D. Coding accuracy of administrative drug claims in the Ontario Drug Benefit database. The Canadian journal of clinical pharmacology = Journal canadien de pharmacologie clinique. 2003;10(2):67-71.
3. Chiu M, Lebenbaum M, Lam K et al. Describing the linkages of the immigration, refugees and citizenship Canada permanent resident data and vital statistics death registry to Ontario's administrative health database. BMC Med Inform Decis Mak. Oct 21 2016;16(1):135.
4. Kadri SS, Gundrum J, Warner S et al. Uptake and Accuracy of the Diagnosis Code for COVID-19 Among US Hospitalizations. Jama. Dec 22 2020;324(24):2553-2554
5. Widdifield J, Bombardier C, Bernatsky S, Paterson JM, Green D, Young J, Ivers N, Butt DA, Jaakkimainen RL, Thorne JC, Tu K. An administrative data validation study of the accuracy of algorithms for identifying rheumatoid arthritis: the influence of the reference standard on algorithm performance. BMC musculoskeletal disorders. 2014 Jun 23;15(1):216.
6. Widdifield J, Bernatsky S, Paterson JM, Tu K, Ng R, Thorne JC, Pope JE, Bombardier C. Accuracy of Canadian health administrative databases in identifying patients with rheumatoid arthritis: a validation study using the medical records of rheumatologists. Arthritis care & research. 2013 Oct 1;65(10):1582-91.
7. Gershon AS, Wang C, Guan J, Vasilevska-Ristovska J, Cicutto L, To T. Identifying patients with physician-diagnosed asthma in health administrative databases. Can Respir J 2009;16:183-8.
8. Andrea S. Gershon, Jun Guan, Chengning Wang, Teresa To; Trends in Asthma Prevalence and Incidence in Ontario, Canada, 1996–2005: A Population Study. Am J Epidemiol 2010; 172 (6): 728-736.
9. To T, Dell S, Dick P, et al. Defining asthma in children for surveillance, Am J Respir Crit Care Med, 2004, vol. 169 7pg. A383.
10. Schultz SE, Rothwell DM, Chen Z, Tu K. Identifying cases of congestive heart failure from administrative data: a validation study using primary care patient records. Chronic diseases and injuries in Canada 2013;33:160-6.
11. Gershon A, Wang C, Guan J, Vasilevska-Ristovska J, Cicutto L, To T. Identifying individuals with physician diagnosed COPD in health administrative databases. COPD 2009;6:388-94.
12. Austin P, Daly PA, Tu JV. A multicenter study of the coding accuracy of hospital discharge administrative data for patients admitted to cardiac care units in Ontario. American Heart Journal 2002; 144(2): 290-6. doi:10.1067/mhj.2002.123839.
13. Guttmann A, Nakhla M, Henderson M, To T, Daneman D, Cauch-Dudek K, Wang X, Lam K, Hux J. Validation of a health administrative data algorithm for assessing the epidemiology of diabetes in Canadian children. Pediatric diabetes. 2010 Mar 1;11(2):122-8.
14. Hux JE, Ivis F, Flintoft V, Bica A. Diabetes in Ontario Determination of prevalence and incidence using a validated administrative data algorithm. Diabetes care 2002;25:512-6.
15. Tony Antoniou, Brandon Zagorski, Mona R. Loutfy, Carol Strike, Richard H. Glazier. Validation of Case-Finding Algorithms Derived from Administrative Data for Identifying Adults Living with Human Immunodeficiency Virus Infection. Plos One. 2011;6(6):e21748. Epub 2011 Jun 30.
16. Tu K, Campbell NR, Chen Z-L, Cauch-Dudek KJ, McAlister FA. Accuracy of administrative databases in identifying patients with hypertension. Open Medicine 2007;1:18-26.
17. Tu K, Chen Z, Lipscombe LL, Canadian Hypertension Education Program Outcomes Research Taskforce. Prevalence and incidence of hypertension from 1995 to 2005: a population-based study. Canadian Medical Association Journal. 2008 May 20;178(11):1429-35.
18. Eric I. Benchimol, Astrid Guttmann, Anne M. Griffiths, Linda Rabeneck, David R. Mack, Herbert Brill, John Howard, Jun Guan, Teresa To, Increasing incidence of paediatric inflammatory bowel disease in Ontario, Canada: evidence from health administrative data, GUT, 2009; 58(11): 1490-1497.
19. Eric I. Benchimol, Astrid Guttmann, David R Mack, Geoffrey C Nguyen, John K Marshall, James C Gregor, Jenna Wong, Alan J Forster, Douglas G Manuel, Validation of international algorithms to identify adults with inflammatory bowel disease in health administrative data from Ontario, Canada, J Clin Epidemiol. 2014; 67(8):887-96.

© 2023 Reppas-Rindlisbacher C et al. *JAMA Network Open*.
