# Supplementary material for: Trends in Delirium and New Antipsychotic and Benzodiazepine Use Among Hospitalized Older Adults Before and After the Onset of the COVID-19 Pandemic
Source: JAMA Netw Open. 2023 Aug 7;6(8):e2327750. doi: 10.1001/jamanetworkopen.2023.27750 (PMC10407689; doi:10.1001/jamanetworkopen.2023.27750)
Supplement: Supplement 2. — Data Sharing Statement [file jamanetwopen-e2327750-s002.pdf]

Data Sharing Statement

Reppas-Rindlisbacher. Trends in Delirium and New Antipsychotic and Benzodiazepine Use Among Hospitalized Older Adults Before and After the Onset of the COVID-19 Pandemic. *JAMA Netw Open*. Published August 07, 2023. doi:10.1001/jamanetworkopen.2023.27750

Data
Data available: No

Additional Information
Explanation for why data not available: The dataset from this study is held securely in coded form at ICES. While data sharing agreements prohibit ICES from making the dataset publicly available, access may be granted to those who meet pre-specified criteria for confidential access, available at https://www.ices.on.ca/DAS. The full dataset creation plan and underlying analytic code are available from the authors upon request, understanding that the computer programs may rely upon coding templates or macros that are unique to ICES and are therefore either inaccessible or may require modification.
